# Unprovoked Thrombosis in a Young Male Revealing a Rare Coexistence of Antiphospholipid Syndrome and Double Heterozygous MTHFR Mutation With Hyperhomocysteinemia

**DOI:** 10.7759/cureus.90260

**Published:** 2025-08-16

**Authors:** Iswarya Lakshme, Avinash C, N Senthil, Viswanathan Pandurangan

**Affiliations:** 1 Internal Medicine, Sri Ramachandra Institute of Higher Education and Research, Chennai, IND; 2 General Medicine, Sri Ramachandra Institute of Higher Education and Research, Chennai, IND

**Keywords:** antiphospholipid syndrome, deep vein thrombosis, double heterozygous mthfr mutation, hypercoagulable state, pulmonary thromboembolism

## Abstract

A case of acute pulmonary thromboembolism (PTE) in a 39-year-old male who presented with progressive dyspnea and left lower limb pain. Imaging studies confirmed extensive pulmonary and deep venous thrombosis. A hypercoagulability workup revealed primary antiphospholipid syndrome (APS) with positive lupus anticoagulant and beta-2 glycoprotein antibodies, along with a compound heterozygous methylenetetrahydrofolate reductase (MTHFR) mutation and hyperhomocysteinemia. This case highlights the significant thrombotic risk posed by the co-occurrence of APS and the compound heterozygous MTHFR mutation. Early identification of such overlapping thrombophilic conditions is crucial for prompt management and effective secondary prevention.

## Introduction

Antiphospholipid syndrome (APS) is an acquired autoimmune thrombophilia characterized by recurrent arterial and/or venous thrombotic events in the presence of persistently elevated anti-phospholipid antibodies (APLA). The autoantibodies are lupus anticoagulant (LA), anticardiolipin antibodies (aCL), and anti-β2-glycoprotein I (aβ2GPI) antibodies [1], of which LA has the highest predisposition to cause vascular events. Venous thrombosis, particularly deep vein thrombosis (DVT) and pulmonary embolism (PE), is a common manifestation; arterial events are most frequently observed affecting cerebral circulation. [2].

The homozygous methylenetetrahydrofolate reductase (MTHFR) gene mutation, which causes hyperhomocysteinemia, has been implicated in various thrombotic disorders [3]. While both conditions individually predispose to thrombosis, their coexistence is very rare and poses a significant additive thrombotic risk. This report details a case of acute pulmonary thromboembolism in a young male with the unusual combination of APS and a heterozygous MTHFR mutation, emphasizing the importance of a comprehensive hypercoagulability workup in unexplained thrombotic events.

## Case presentation

A 39-year-old male, a hotel manager with a history of chronic alcohol use for a period of 10 years, who consumes 180ml/day and is a non-smoker, presented to the emergency department with acute onset chest pain and dyspnea of New York Heart Association (NYHA) class IV of two days duration. He also reported left calf pain for two days. There was no history of fever, palpitations, giddiness, or pedal edema, no history of syncope or hemoptysis, and no history of any sicca symptoms. The patient denied any recent history of surgery, trauma or immobilization. There was no significant family history and no history of any recent vaccination.

On examination, the patient was tachycardic. Vitals recorded were blood pressure 100/70mmHg, pulse rate 104/minute, peripheral oxygen saturation in ambient air 92%. The patient was started on oxygen support at 2 liters/minute via nasal cannula. Systemic examination was unremarkable. Local examination showed left lower limb swelling extending up to the thigh associated with calf tenderness. Peripheral pulses were palpable in bilateral lower limbs.

An electrocardiogram showed sinus tachycardia. An echocardiogram revealed a dilated right atrium and right ventricle with an ejection fraction of 55%. His mean pulmonary artery pressure was 43mmHg and he was admitted to the Intensive Care Unit (ICU) for further evaluation.

The baseline investigations revealed macrocytosis, elevated D-dimer and prolonged activated partial thromboplastin time (aPTT) (Table 1). A provisional diagnosis of pulmonary thromboembolism (PTE) was made and the patient was initiated on intravenous unfractionated heparin 5000 IU every six hours. Renal and liver function tests and complements C3, C4 were within normal levels. Anti-nuclear antibody was positive (nuclear speckled pattern).

**Table 1:** baseline investigations

Test Parameter	Result	Normal Reference Range
Hemoglobin	14 g/dL	Males: 13–18 g/dL; Females: 12–16 g/dL
Mean Corpuscular Volume (MCV)	117 fL	80–100 fL
Platelet Count	112 × 10⁹/L	150–400 × 10⁹/L
HbA₁c	5.5%	4.0–5.9%
D-dimer	6.9 mg/dL	<0.5 mg/L (or <500 ng/mL)
Prothrombin Time (PT)	13.4 seconds	11–12.5 seconds
International Normalized Ratio (INR)	1.1	0.8–1.1
Activated Partial Thromboplastin Time (aPTT)	49 s	30–40 s
Vitamin B₁₂	<200 pg/mL	200–900 pg/mL
Creatinine	0.7 mg/dL	Males: 0.7–1.3 mg/dL; Females: 0.6–1.1 mg/dL
Peripheral Smear	Macrocytosis	Normal: Normocytic, normochromic cells

**Figure 1:**
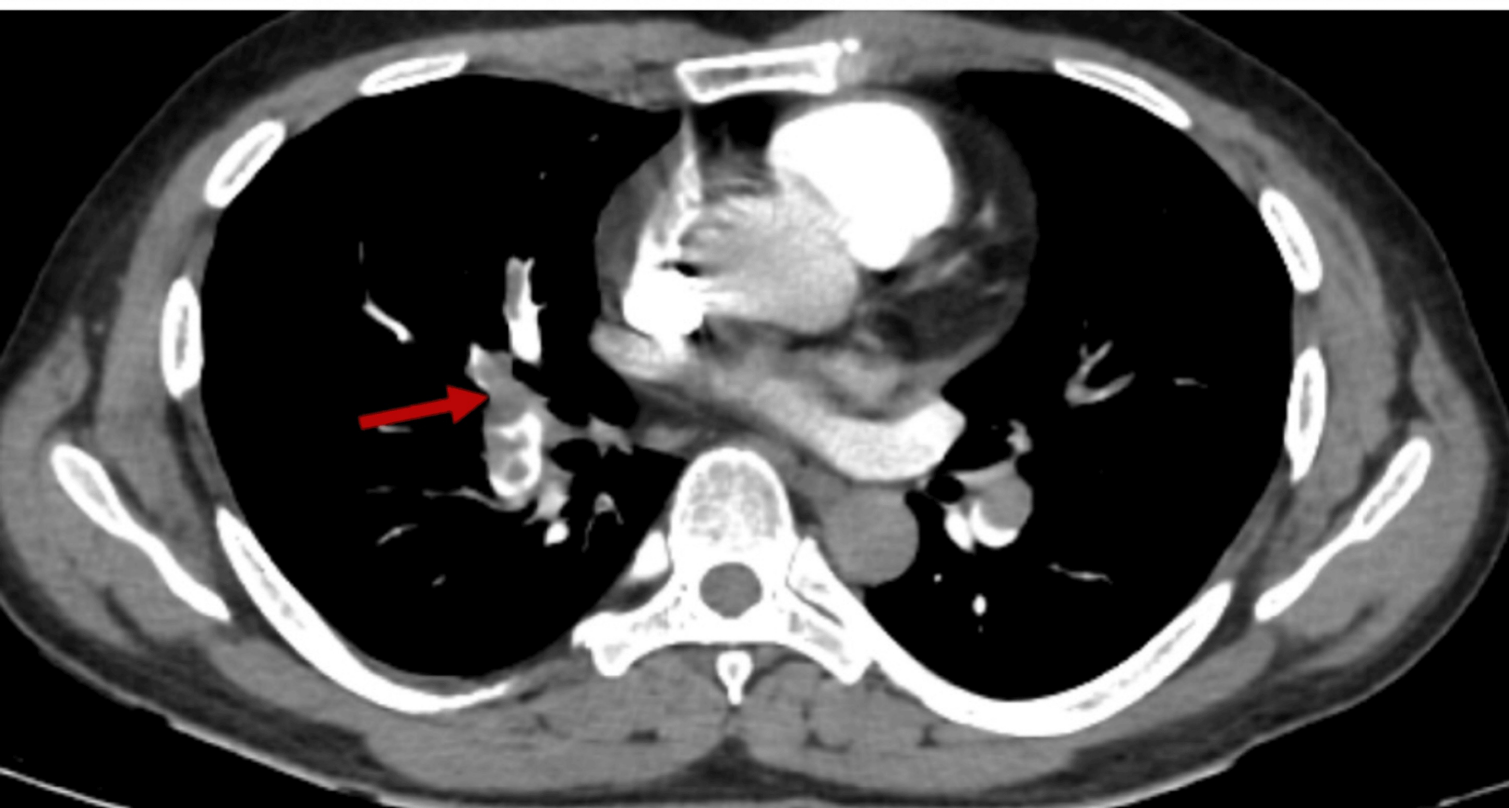
Near complete filling defect in distal portion of right pulmonary artery

**Figure 2:**
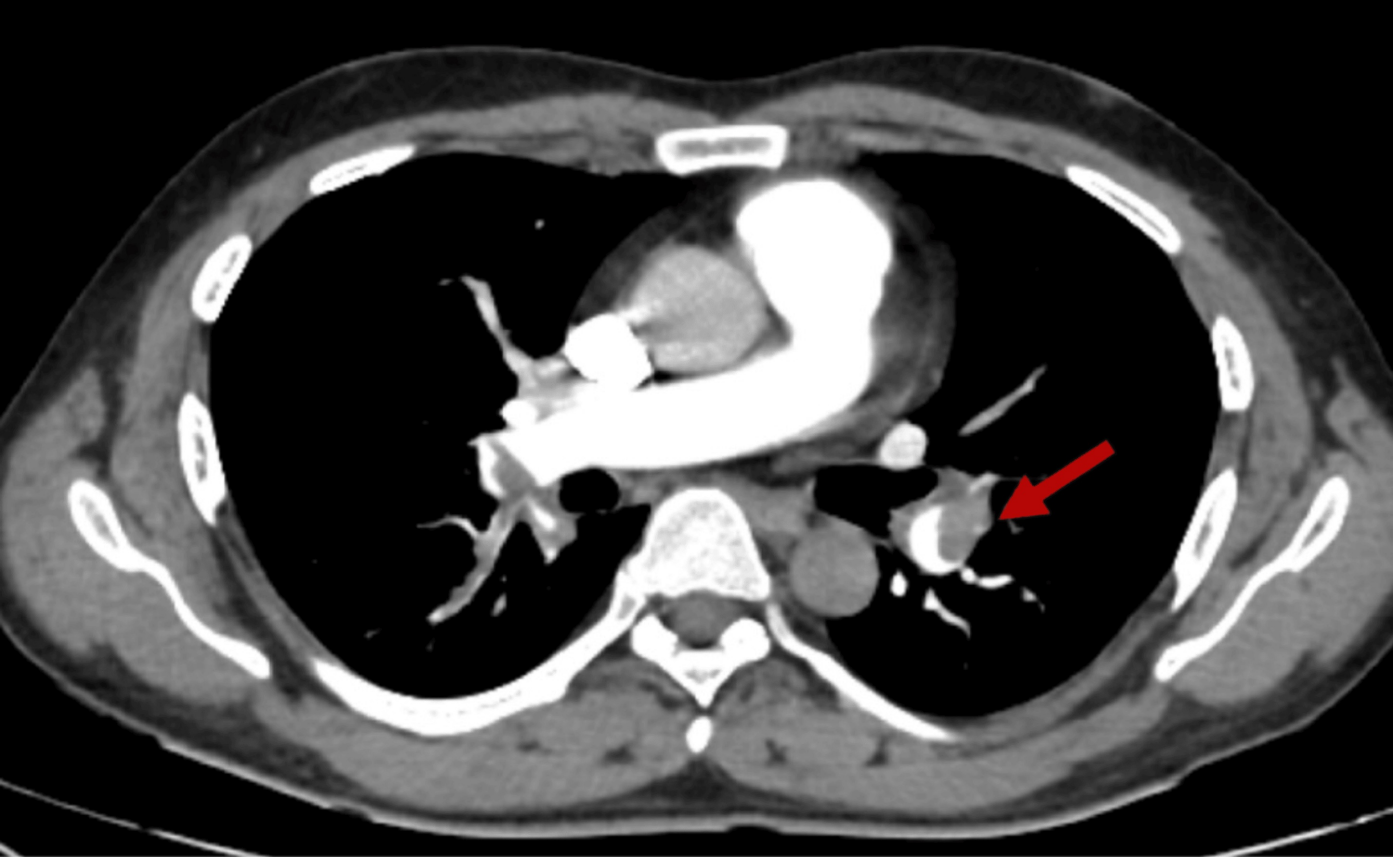
Partial filling defect in distal left main pulmonary artery

Doppler ultrasound of the left lower limb revealed DVT involving the distal superficial femoral, popliteal, and anterior tibial veins and Computed Tomography (CT) pulmonary angiogram was performed, which confirmed a complete filling defect in the right main pulmonary artery and a partial defect in the left main pulmonary artery (Figures 1, 2).

In view of unprovoked thrombosis as evidenced by DVT and PTE at a young age, a comprehensive hypercoagulability workup was performed. Tests revealed aβ2GPI antibodies positive along with LA positive and significantly elevated homocysteine levels. Genetic testing confirmed the presence of a compound double heterozygous mutation of the MTHFR gene (Table 2).

**Table 2:** Hypercoagulability workup APLA: antiphospholipid antibody

PARAMETER	RESULT
APLA profile	1) β₂Glycoprotein I antibodies (IgG-22) positive 2) Lupus anticoagulant positive (detected by diluted Russel Viper Venom Time (dVRRT)) 3) cardiolipin antibodies - negative.
Homocysteine	59.2 µmol/L (elevated) normal 5-15 µmol/L
Vitamin B₁₂	Deficient (<200 pg/dl)
MTHFR mutation	Compound double heterozygous detected - CT677T and AC1298c
Anti-nuclear antibody	Positive - nuclear speckled pattern

Other investigations, including double-stranded DNA and direct Coombs test, were negative.

A diagnosis of APS with coexistent hyperhomocysteinemia due to compound heterozygous MTHFR mutation and B12 deficiency was made. Hence our patient's unprovoked thrombophilic state was attributed to positivity of LA and aβ2GPI antibodies, compound double heterozygous MTHFR mutation, and severe hyperhomocystinemia.

Heparin was subsequently bridged with oral anticoagulants: tablet warfarin 2mg was given once daily at evening for a duration of three days and subsequently stopped later. Anticoagulation with warfarin, vitamin B12 1000mcg intravenous and folate supplementation were initiated. The patient was discharged on long-term oral anticoagulation (international normalized ratio (INR) at discharge was 1.6) and advised regarding regular INR monitoring and to restrict green leafy vegetables.

## Discussion

APS is recognized as an acquired autoimmune thrombophilia, characterized by recurrent thrombotic events. It has been shown that APLA can disrupt natural anticoagulant pathways, such as the protein C system, and upregulate tissue factor expression, thereby further promoting coagulation. The MTHFR gene encodes for methylenetetrahydrofolate reductase, an enzyme critical in folate metabolism that catalyzes the conversion of 5,10-methylenetetrahydrofolate to 5-methyltetrahydrofolate. This latter compound is essential for the remethylation of homocysteine to methionine. Common polymorphisms, such as the C677T variant, are known to lead to a thermolabile enzyme with reduced activity (approximately 50% for heterozygotes and lower for homozygotes), resulting in elevated homocysteine levels, referred to as hyperhomocysteinemia [3,4].

Hyperhomocysteinemia itself has been recognized as a risk factor for both arterial and venous thromboembolism, including pulmonary embolism. Elevated homocysteine levels are thought to induce endothelial cell damage and cause alteration between procoagulant and anticoagulant factors, thereby contributing to a prothrombotic state. The risk is generally considered to be proportional to the degree of homocysteine elevation [5].

While both APS and MTHFR mutations independently predispose to thrombosis, their co-occurrence, as was observed in the presented patient, is considered to lead to an increased prothrombotic risk. Although the MTHFR mutation alone, especially in the homozygous state, is an independent risk factor [6], the heterozygous state is not significantly associated with increased risk of thrombotic state. However patients carrying a double heterozygous mutation as seen in our patient independent of other risk factors possess significant risk factor and its presence in conjunction with a strong positivity of APS is postulated to create a synergistic effect, potentially overcoming the body's natural anticoagulant mechanisms and leading to severe thrombotic episodes.

The presented patient, a 39-year-old male, was observed to have acute, extensive pulmonary and deep venous thrombosis without provoking factors. The diagnosis of primary APS, confirmed by positive LA and aβ2GPI antibodies, was identified as a major underlying cause for his thrombotic event. The subsequent discovery of a heterozygous MTHFR mutation and significant hyperhomocysteinemia further elucidated the multifactorial risk nature of his thrombotic predisposition.

This case highlights the importance of a comprehensive hypercoagulability workup in young individuals presenting with unprovoked thrombosis, as it unravels the identification of multiple thrombophilic risk factors which has an impact on therapeutic decision.

## Conclusions

This case emphasizes the clinical significance of concurrent prothrombotic disorders in the etiology of severe venous thromboembolic events. The presence of both APS and compound double heterozygous MTHFR mutation may act synergistically to increase the thrombotic risks. Such dual pathology can predispose even young individuals to life-threatening complications. Recognizing and addressing multiple contributing mechanisms is essential to guide optimal therapeutic strategies and to prevent recurrence. Hence treating physicians must be aware of the overlapping risk factors that can be identified as in a single patient.
